# Supplementary material for: The sensor of the bacterial histidine kinase CpxA is a novel dimer of extracytoplasmic Per-ARNT-Sim domains
Source: J Biol Chem. 2024 Apr 4;300(5):107265. doi: 10.1016/j.jbc.2024.107265 (PMC11078701; doi:10.1016/j.jbc.2024.107265)
Supplement: Supporting Information [file mmc1.pdf]

Table S1. Data collection and refinement statistics

	CpxA (31-163)	CpxA (31-163) †
**Wavelength**	1.03316	0.97950
**Resolution range**	39.2-1.8 (1.86-1.8)	39.6-2.0 (2.07-2.0)
**Space group**	P 21 21 21	P 21 21 21
**Unit cell**	37.16 43.16 186.98 90 90 90	37.05 43.77 186.34 90 90 90
**Total reflections**	227024 (21825)	170877 (5615)
**Unique reflections**	28482 (2769)	21058 (1864)
**Multiplicity**	8.0 (7.9)	8.1 (3.0)
**Completeness (%)**	98.1 (96.6)	98.4 (89.0)
**Mean I/sigma(I)**	13.75 (2.68)	13.83 (1.37)
**Wilson B-factor**	34.85	34.28
**R-merge**	0.070 (1.09)	0.078 (0.800)
**R-meas**	0.076 (1.16)	0.083 (0.926)
**R-pim**	0.028 (0.408)	0.027 (0.488)
**CC 1/2**	0.997 (0.958)	0.999 (0.741)
**CC***	0.999 (0.989)	1 (0.923)
**Reflections used in refinement**	28337 (2735)	21045 (1861)
**Reflections used for R-free**	1345 (133)	969 (86)
**R-work**	0.204 (0.303)	
**R-free**	0.247 (0.348)	
**CC(work)**	0.955 (0.919)	
**CC(free)**	0.954 (0.856)	
**Number of non-hydrogen atoms**	2032	
macromolecules	1943	
solvent	89	
**Protein residues**	233	
**RMS(bonds)**	0.013	
**RMS(angles)**	1.15	
**Ramachandran favored (%)**	97.82	
**Ramachandran allowed (%)**	2.18	
**Ramachandran outliers (%)**	0.00	
**Rotamer outliers (%)**	2.91	
**Clashscore**	3.10	
**Average B-factor**	56.92	
macromolecules	57.16	
solvent	51.77	
**Number of TLS groups**	16	

Statistics for the highest-resolution shell are shown in parentheses.

† Used for phasing.

Table S2. Strains used in this study.

Strain	Description	Source
MC4100	F_ *araD139* (*argF-lac*)*U169 rpsL150* (Strr) *relA1 flbB5301 decC1 ptsF25 rbsR*	(1)
TR50	MC4100 λRS88[*cpxP'-lacZ*$^+$]	(2)
RM53	TR50 Δ*cpxA*	This study
GLT100	BL21(DE3) + pGEX-*cpxA*$_{31-163}$	This study
RM336	MC4100 *cpxA*$_{N107A}$	This study
RM367	MC4100 *cpxA*$_{K121A}$	This study
RM338	MC4100 *cpxA*$_{Y123A}$	This study
RM441	MC4100 IRS88[*cpxP-lacZ*] *cpxA*$_{N107A}$	This study
RM448	MC4100 IRS88[*cpxP-lacZ*] *cpxA*$_{K121A}$	This study
RM444	MC4100 IRS88[*cpxP-lacZ*] *cpxA*$_{Y123A}$	This study
RM477	RM441 + pBR322	This study
RM478	RM441 + pLD404	This study
RM481	RM448 + pBR322	This study
RM482	RM448 + pLD404	This study
RM483	RM444 + pBR322	This study
RM484	RM444 + pLD404	This study
TC636	RM53 + pK184	This study
RMQ2	RM53 + pK184-*cpxA*$_{WT}$	This study
RMQ6	RM53 + pK184-*cpxA*$_{M48K}$	This study
RMQ7	RM53 + pK184-*cpxA*$_{D113K}$	This study
RMQ21	RM53 + pK184-*cpxA*$_{WT}$ + pCA24N	This study
RMQ22	RM53 + pK184-*cpxA*$_{WT}$ + pCA-*nlpE*	This study
RMQ23	RM53 + pK184-*traJ* + pCA24N	This study
RMQ24	RM53 + pK184-*traJ* + pCA-*nlpE*	This study
RMQ27	RM53 + pK184-*cpxA*$_{M48K}$ + pCA24N	This study
RMQ28	RM53 + pK184-*cpxA*$_{M48K}$ + pCA-*nlpE*	This study
RMQ29	RM53 + pK184-*cpxA*$_{D113K}$ + pCA24N	This study
RMQ30	RM53 + pK184-*cpxA*$_{D113K}$ + pCA-*nlpE*	This study
RMQ34	RM53 + pK184-*cpxA*$_{E91A}$	This study
RMQ52	RM53 + pK184-*cpxA*$_{E91A}$ + pCA24N	This study
RMQ53	RM53 + pK184-*cpxA*$_{E91A}$ + pCA-*nlpE*	This study
TC643	RM53 + pK184-*cpxA*$_{E91K}$	This study
TC644	RM53 + pK184-*cpxA*$_{E91K+R93E}$	This study
TC646	RM53 + pK184 + pTrc99A	This study
TC647	RM53 + pK184 + pTrc-*nlpE*	This study
TC648	RM53 + pK184-*cpxA* + pTrc99A	This study
TC649	RM53 + pK184-*cpxA* + pTrc-*nlpE*	This study
TC650	RM53 + pK184-*cpxA*$_{E91K}$ + pTrc99A	This study
TC651	RM53 + pK184-*cpxA*$_{E91K}$ + pTrc-*nlpE*	This study
TC652	RM53 + pK184-*cpxA*$_{E91K+R93E}$ + pTrc99A	This study
TC653	RM53 + pK184-*cpxA*$_{E91K+R93E}$ + pTrc-*nlpE*	This study
TC726	RM53 + pK184-*cpxA*$_{N107D}$	This study
TC719	RM53 + pK184-*cpxA*$_{Q103E}$	This study
TC721	RM53 + pK184-*cpxA*$_{R106E}$	This study
TC758	RM53 + pK184-*cpxA*$_{N107D}$ + pTrc99A	This study
TC761	RM53 + pK184-*cpxA*$_{N107D}$ + pTrc-*nlpE*	This study
TC763	RM53 + pK184-*cpxA*$_{Q103E+D113N}$	This study
TC756	RM53 + pK184-*cpxA*$_{Q103E}$ + pTrc99A	This study
TC759	RM53 + pK184-*cpxA*$_{Q103E}$ + pTrc-*nlpE*	This study
TC757	RM53 + pK184-*cpxA*$_{R106E}$ + pTrc99A	This study
TC760	RM53 + pK184-*cpxA*$_{R106E}$ + pTrc-*nlpE*	This study

TC724	RM53 + pK184-*cpxA*$_{E91K+R99E}$	This study
TC725	RM53 + pK184-*cpxA*$_{E91K+R93E+R99E}$	This study
TC796	RM53 + pK184-*cpxA*$_{E91K+R99E}$ + pTrc99A	This study
TC797	RM53 + pK184-*cpxA*$_{E91K+R99E}$ + pTrc-*nlpE*	This study
TC798	RM53 + pK184-*cpxA*$_{E91K+R93E+R99E}$ + pTrc99A	This study
TC799	RM53 + pK184-*cpxA*$_{E91K+R93E+R99E}$ + pTrc-*nlpE*	This study

Table S3. Primers used in this study.

Primer Name	Sequence (5'-3')	Notes
For generation of cpxA *chromosomal mutants*		
pCAF8	CGTCTTCACCTCGAGAAATC	Anchor primer to pCA24N encoding SfiI sites
pCAR4	TTGCATCACCTTCACCCTCTCCACTGACAG	Anchor primer to pCA24N encoding SfiI sites
CpxAN$_{107}$AFw	AACTCTTCAGCTTTTATTGGTCAGGCCGA	Mutagenic primer to generate N107A fragment with EarI site
CpxAN$_{107}$ARv	AACTCTTCAAGCACGAATGATCTGCATTTCG	Mutagenic primer to generate N107A fragment with EarI site
CpxAK$_{121}$AFw	AACTCTTCAGCTAAGTATGGCCGCGTGGA	Mutagenic primer to generate K121A fragment with EarI site
CpxAK$_{121}$ARv	AACTCTTCAAGCCTTCTGCGGATGATCGG	Mutagenic primer to generate K121A fragment with EarI site
CpxAY$_{123}$AFw	AACTCTTCAGCAGGCCGCGTGGAACTGGT	Mutagenic primer to generate Y123A fragment with EarI site
CpxAY$_{123}$ARv	AACTCTTCATGCCTTTTTCTTCTGCGGAT	Mutagenic primer to generate Y123A fragment with EarI site
cpxA-cat	ATTTAATGTGGTGGCGGCGTCTGTTCCGGGCGATTG ATAAGTGGGCACCGTGTGACGGAAGATCACTTCGCAG	For amplification of *cat-sacB* cassette
sacB-cpxA	GGTCAAACAGTAAGTTAATGAAATCGGATTGAGAA CTGCTGGCCGGATCAAAGGGAAAACTGTCCATAT	For amplification of *cat-sacB* cassette
*cpxA*181Fw	TCCGCCCAACGATTTAATG	For amplification of *cpxA* variants from pCA24N expression vector
*cpxA*503Rv	AGCAGTAATAGCGGGCGGT	For amplification of *cpxA* variants from pCA24N expression vector
For generation of CpxA$_{31-163}$ expression vector		
5'-31	CGCGGATCCGATTCACGCCAGATGACCGA	For amplification of *cpxA*$_{31-163}$ from genome
3'-163	GCGAATTCCTAGCGGTCAAACAGTAAGTT	For amplification of *cpxA*$_{31-163}$ from genome
*For generation of pK184-*cpxA		
XA-1 fw	ATAGGATCCGTGAGGAGGTTCCTATGATAGGCAGCTTAACCGCGC	For amplification of *cpxA* with EcoRI site (and for creation of overlap fragments, see below)
XA-2c rv	TATATAAGCTTCTGCAGTTATGACCGCTTATACAGCGGCAACCAAATCACC	For amplification of *cpxA* with BamHI site
pK184_F	CGTATGTTGTGTGGAATTGTG	For sequencing of inserts into pK184
pK184_R	CAAGGCGATTAAGTTGGGTAA	For sequencing of inserts into pK184
*For generation of mutations in pK184-*cpxA		
XA-2b rv	GGCAAGGAATTCCTGTGGCCC	For generation of downstream fragment for all mutations
XA-5 fw	AGATTGAGCAGCATGTCGAAGCG	For generation of downstream fragment (with XA-2b) to create M48K mutation by overlap extension PCR
XA-6 rv	CGCTTCGACATGCTGCTCAATCTTCAGACCCTGACGCTGTTCGCT	For generation of upstream fragment (with XA-1) to create M48K mutation by overlap extension PCR

XA-7 fw	AAAAACGCCGATCATCCGCAGAAG	For generation of downstream fragment (with XA-2b) to create D113K mutation by overlap extension PCR
XA-8 rv	CTTCTGCGATGATCGGCGTTTTTGGCCTGACCAATAAAGTTACGAATGATCTGC	For generation of upstream fragment (with XA-1) to create D113K mutation by overlap extension PCR
CpxA E91A-fw	GGTGACCACCGCTGGCCGCGTGA	For generation of E91A by Q5 site-directed mutagenesis
CpxA E91A-rv	AATAACAAACGCTGTCCTGGC	For generation of E91A by Q5 site-directed mutagenesis
CpxA E91K-fw	GGTGACCACCAAAGGCCGCGTGA	For generation of E91K by Q5 site-directed mutagenesis
CpxA E91K-rv	AATAACAAACGCTGTCCTGGCGG	For generation of E91K by Q5 site-directed mutagenesis
Q5SDM_E91K_R93E_F	CGAAGTGATCGGCGCTGAACGC	For generation of E91K+R93E by Q5 site-directed mutagenesis
Q5SDM_E91K_R93E_R	CCTTTGGTGGTCACCAATAACAAACGC	For generation of E91K+R93E by Q5 site-directed mutagenesis
Q5SDM_99+91_F	CGGCGCTGAAGAAAGCGAAATGCAGATCATTC	For generation of E91K+R99E by Q5 site-directed mutagenesis
Q5SDM_99+91_R	ATCACGCGGCCTTTGGTG	For generation of E91K+R99E by Q5 site-directed mutagenesis
Q5SDM_91 93 99_F	CGGCGCTGAAGAAAGCGAAATGC	For generation of E91K+R93E+R99E by Q5 site-directed mutagenesis
Q5SDM_91 93 99_R	ATCACTTCGCCTTTGGTG	For generation of E91K+R93E+R99E by Q5 site-directed mutagenesis
Q5SDM_Q103E_F	CAGCGAAATGGAAATCATTCGTAACTTTATTG	For generation of Q103E by Q5 site-directed mutagenesis
Q5SDM_Q103E_R	CGTTCAGCGCCGATCACG	For generation of Q103E by Q5 site-directed mutagenesis
Q5SDM_R106E_F	GCAGATCATTGAAAACTTTATTGGTCAGGCC	For generation of R106E by Q5 site-directed mutagenesis
Q5SDM_R106E_R	ATTTCGCTGCGTTCAGCG	For generation of R106E by Q5 site-directed mutagenesis
Q5SDM_N107D_F	GATCATTCGTGATTTTATTGGTCAGGCC	For generation of N107D by Q5 site-directed mutagenesis
Q5SDM_N107D_R	TGCATTTCGCTGCGTTCA	For generation of N107D by Q5 site-directed mutagenesis
Q5SDM_D113N_F	TGGTCAGGCCAATAACGCCGATC	For generation of Q103E+D113N by Q5 site-directed mutagenesis
Q5SDM_103+113_R	ATAAAGTTACGAATGATTTCCATTTCGC	For generation of Q103E+D113N by Q5 site-directed mutagenesis

Table S4. Plasmids used in this study.

Plasmid	Description	Source
pCA24N	Empty ASKA library vector, Cam^R	
pCA-*cpxA*	CpxA expression from pCA24N backbone, IPTG-inducible, ASKA library (GFP-), Cam^R	
pTrc99A	Empty expression vector, IPTG-inducible from *trc* promoter, Amp^R	
pTrc-$nlpE_{WT}$	His-tagged NlpE expression from pTrc99A backbone, IPTG-inducible, Amp^R	
pBR322	Cloning vector, Amp^R	
pLD404	NlpE expression from the pBR322 backbone, Amp^R	
pK184	Empty expression vector, Kan^R	
pK184-*traJ*	pK184 encoding for the *traJ* locus which was used as a less toxic vector control in some experiments as it has no impact on activation of the Cpx response, Kan^R	
pK184-$cpxA_{WT}$	WT *cpxA* cloned into pK184, Kan^R	This study
pK184-$cpxA_{M48K}$	*cpxA* M48K cloned into pK184, Kan^R	This study
pK184-$cpxA_{D113K}$	*cpxA* D113K cloned into pK184, Kan^R	This study
pK184-$cpxA_{E91A}$	*cpxA* E91A cloned into pK184, Kan^R	This study
pK184-$cpxA_{E91K}$	*cpxA* E91K cloned into pK184, Kan^R	This study
pK184-$cpxA_{E91K+R93E}$	*cpxA* E91K+R93E cloned into pK184, Kan^R	This study
pK184-$cpxA_{N107D}$	*cpxA* N107D cloned into pK184, Kan^R	This study
pK184-$cpxA_{Q103E}$	*cpxA* Q103E cloned into pK184, Kan^R	This study
pK184-$cpxA_{R106E}$	*cpxA* R106E cloned into pK184, Kan^R	This study
pK184-$cpxA_{Q103E+D113N}$	*cpxA* Q103E+D113N cloned into pK184, Kan^R	This study
pK184-$cpxA_{E91K+R99E}$	*cpxA* E91K+R99E cloned into pK184, Kan^R	This study
pK184-$cpxA_{E91K+R93E+R99E}$	*cpxA* E91K+R93E+R99E cloned into pK184, Kan^R	This study
pFLP2	Plasmid encoding for Flp recombinase, Amp^R	
pKD46	Plasmid encoding for λRed functions, Amp^R	
pRM24	pCA-$cpxA_{N107A}$, pCA24N vector harbouring *cpxA* N107A	This study
pRM12	pCA-$cpxA_{K121A}$, pCA24N vector harbouring *cpxA* K121A	This study
pRM13	pCA-$cpxA_{Y123A}$, pCA24N vector harbouring *cpxA* Y123A	This study

Table S5. Modeling parameters and outputs of ColabFold.

Model	Avg pLDDT	pTm	Sequences used	# Sequences	Start Res	End Res
CpxA ecoli Dimer_10	87.65	0.76	mmSeqs2 + pHMMER_98%_non-redundant	1214	8	184
CpxA vib	87.09	0.82	mmSeqs2	484	14	195
CpxA ecoli PAS-TM-HAMP	85.69	0.76	mmSeqs2	3647	1	233

A

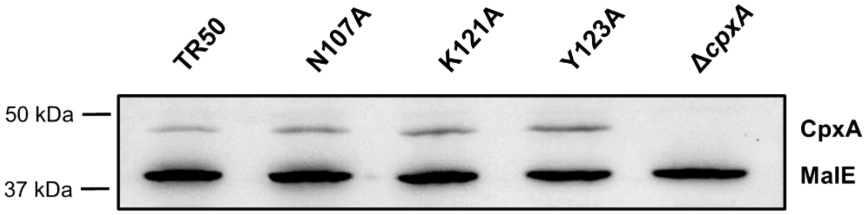

B

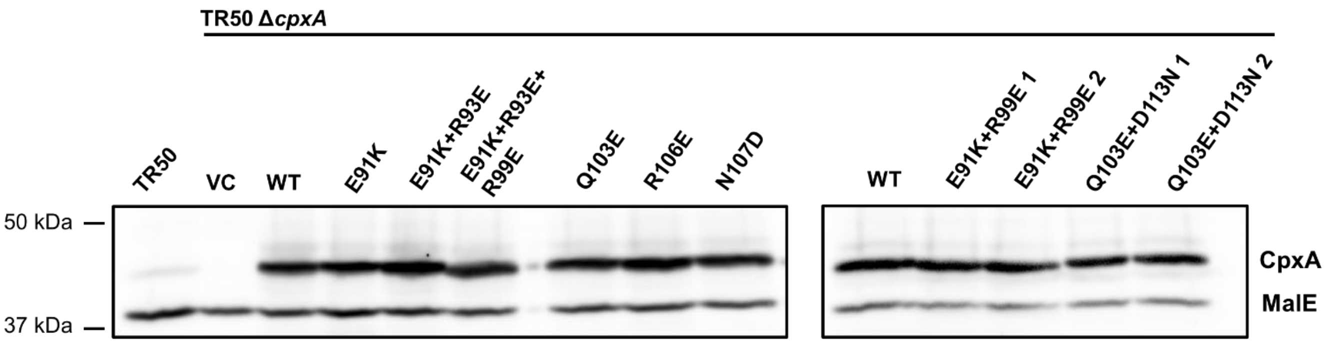

Figure S1. Expression levels of **(A)** *cpxA* chromosomal mutants and **(B)** plasmid-based mutations of CpxA in a Western blot using an anti-CpxA-MBP antibody from whole-cell lysate.

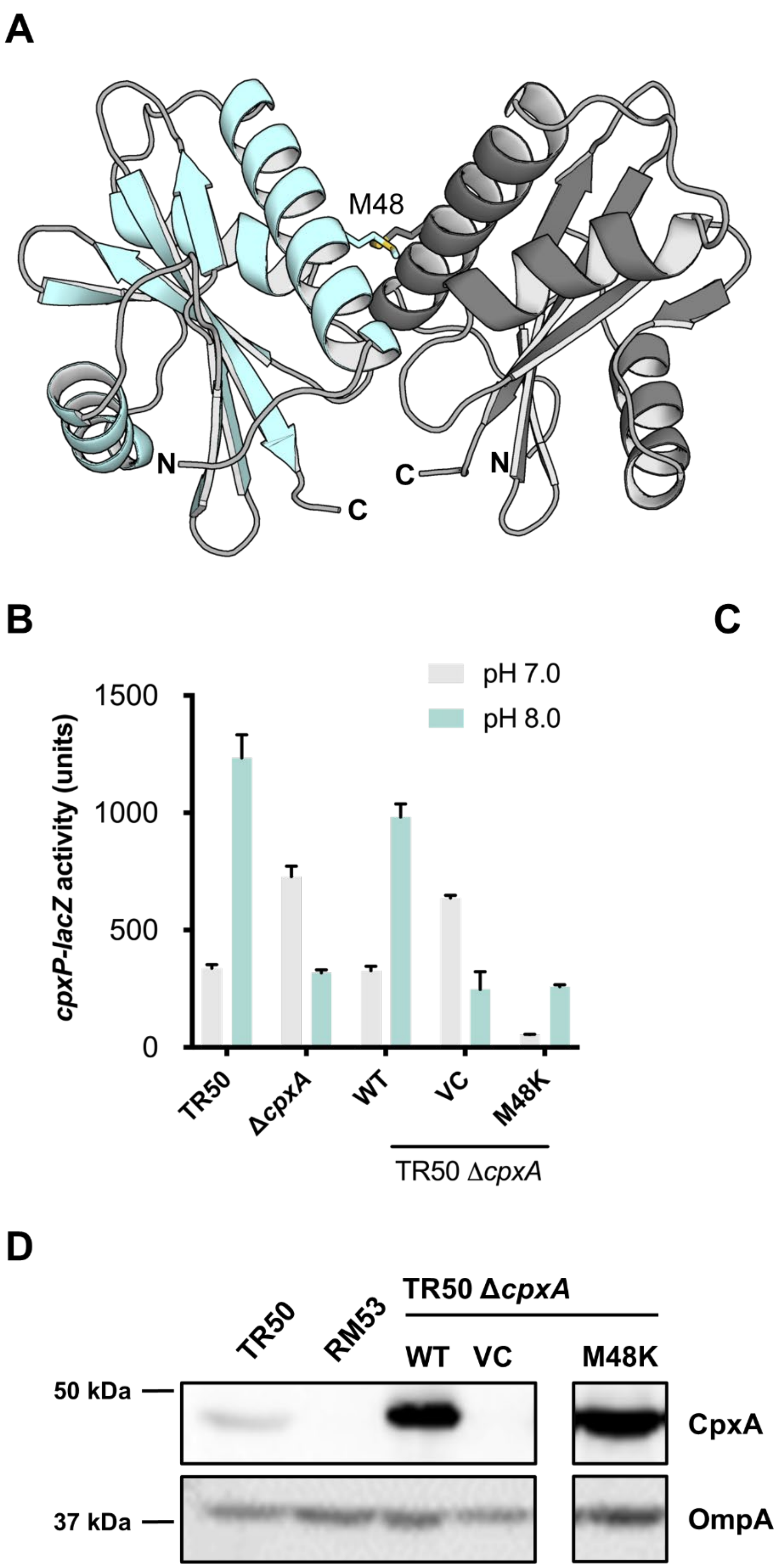

Figure S2. Crystal dimer structure of CpxA. **(A)** Ribbon cartoon diagram of the dimer with each monomer shown in a different color. The main dimer interface residue M48 is highlighted. The ability of the M48K mutation to sense **(B)** alkaline pH and **(C)** NlpE overexpression, as seen in the activity of a *cpxP-lacZ* transcriptional reporter. Shown are mean with standard deviation of three replicates from three independent experiments. **(D)** shows the expression levels of CpxA in relevant strains as determined by Western blotting with anti-CpxA-MBP antibody.

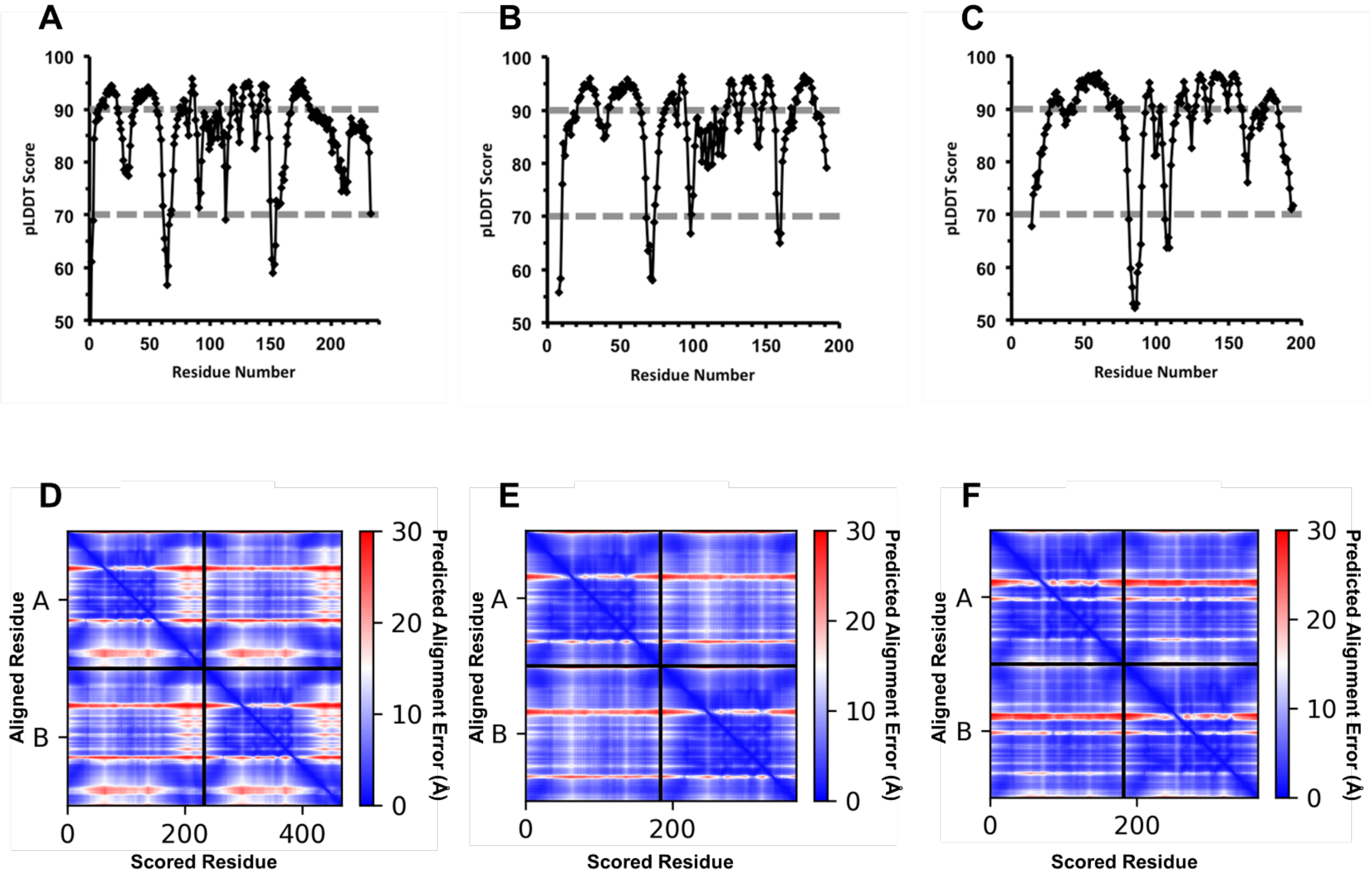

Figure S3. AlphaFold2 confidence metrics for *E. coli* and *V. parahaemolyticus* models. **A-C** Predicted local distance difference test (pLDDT) scores per residue for CpxA-EC TM1-HAMP **(A)**, CpxA-SD$_{EC}$ **(B)**, CpxA-SD$_{Vib}$ **(C)**. Dashed lines indicate cut offs for very high confidence (pLDDT > 90) and high confidence (pLDDT > 70). **D-F** Predicted alignment error (pAE) within monomers (Top Left and Bottom Right sub-panels) and between monomers (Top Right and Bottom Left sub-panels) for CpxA-EC TM1-HAMP **(D)**, CpxA-SD$_{EC}$ **(E)**, CpxA-SD$_{Vib}$ **(F)**.

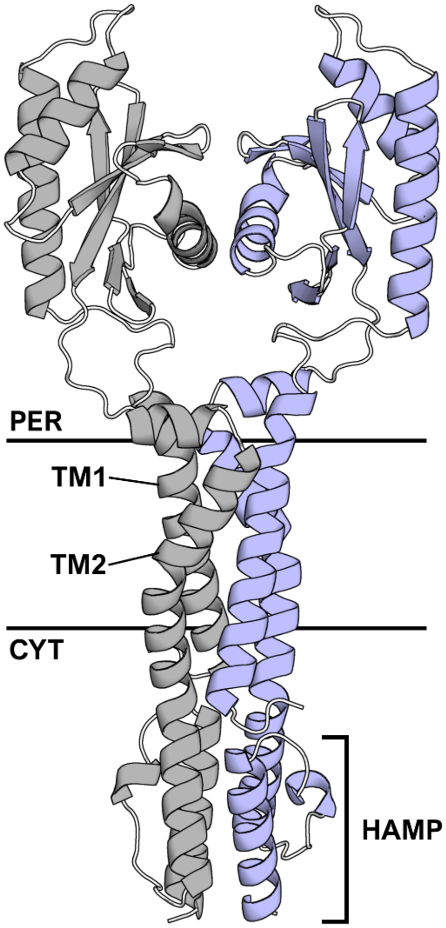

Figure S4. AlphaFold2 model of *E. coli* CpxA including transmembrane domains, periplasmic sensor domains and cytosolic HAMP domains.

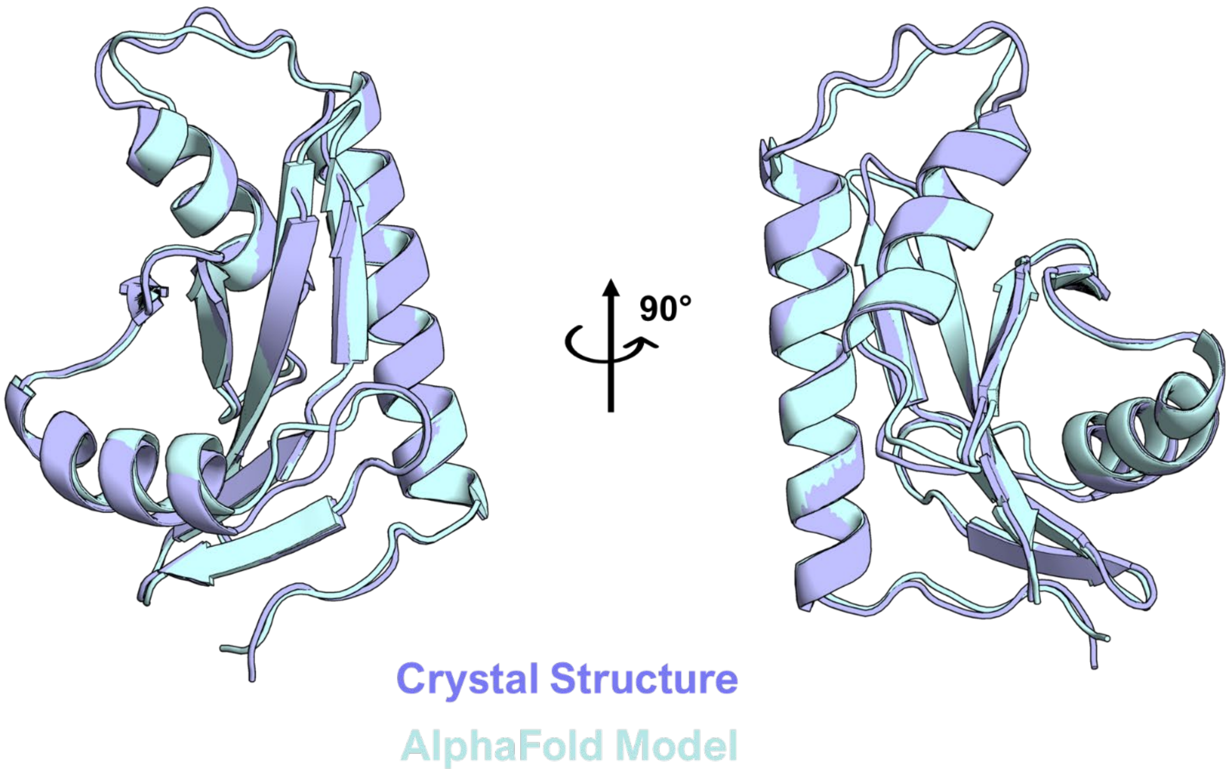

Figure S5. Alignment of the crystal structure and AlphaFold2 model monomer of *E. coli* $CpxA_{SD}$.

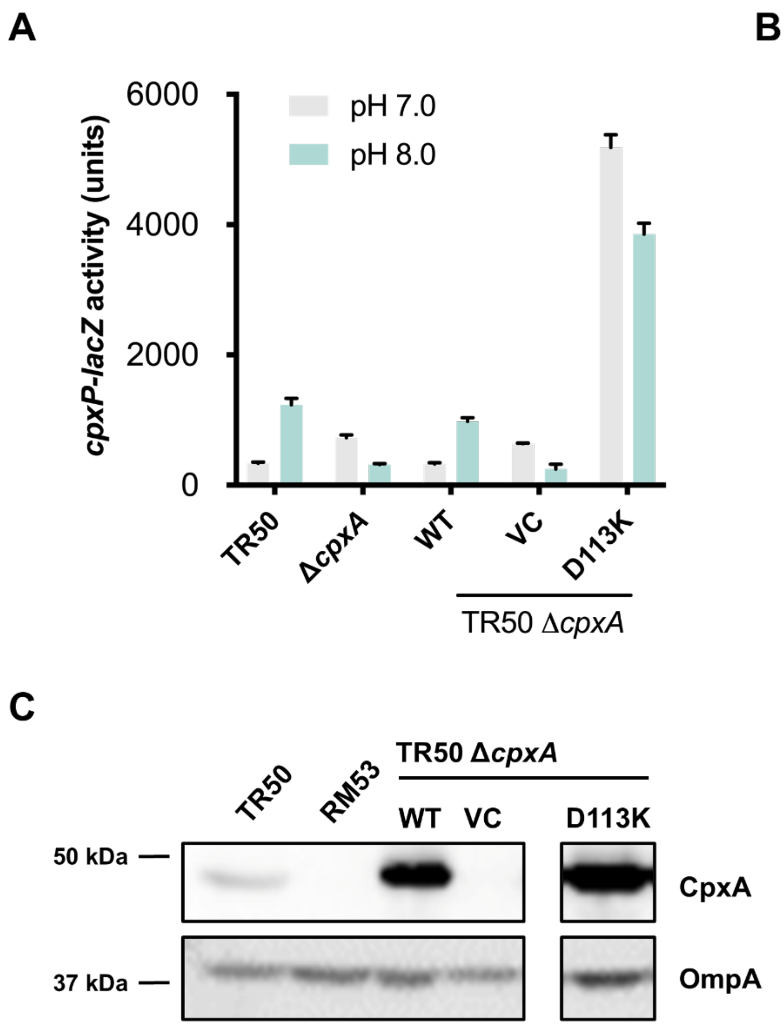

Figure S6. Ability of plasmid-borne CpxA D113K variant to sense **(A)** alkaline pH and **(B)** NlpE overexpression. Shown are mean with standard deviation of three replicates from three independent experiments. **(C)** shows the expression level of D113K compared to WT CpxA by Western blotting.

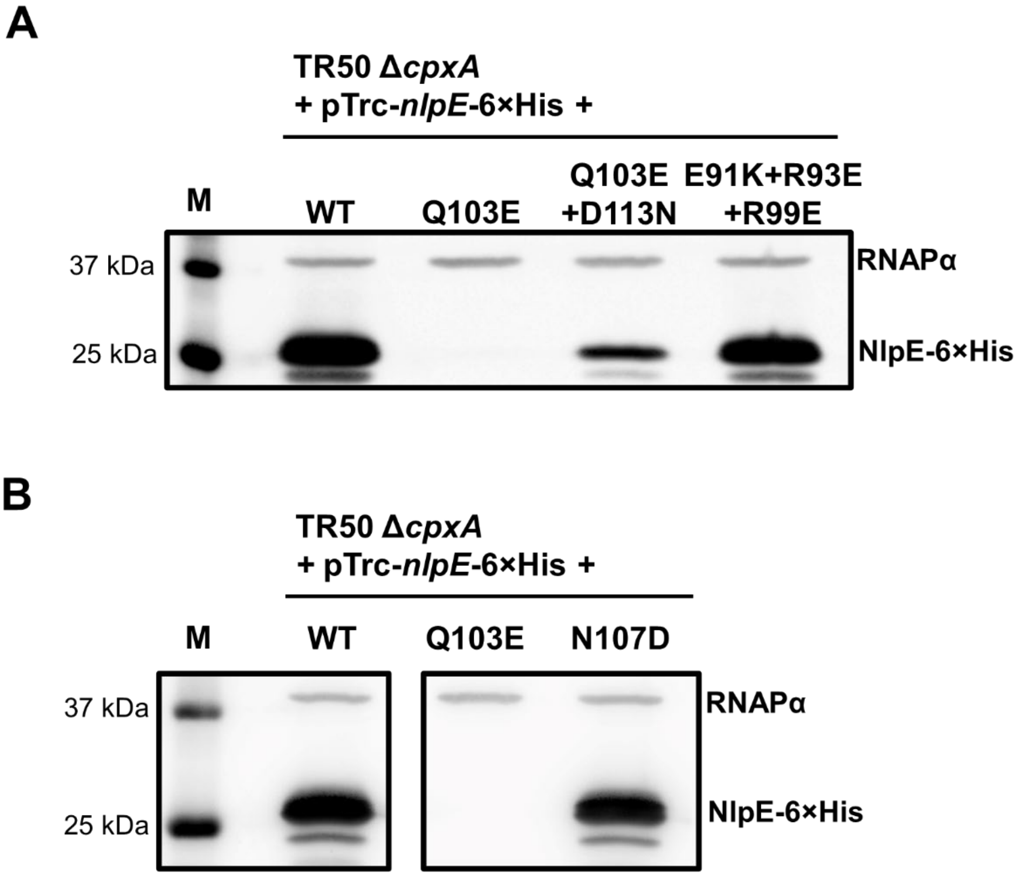

Figure S7. Western blots showing expression levels of His-tagged NlpE in strains expressing CpxA mutants. Strains cultured in identical conditions to reporter assay experiments were harvested, lysed and prepared for SDS-PAGE. After transfer, membranes were probed with antibody raised against RNAP α subunit (loading control) and His×6 (NlpE). The lane labelled "M" contains the molecular weight markers.

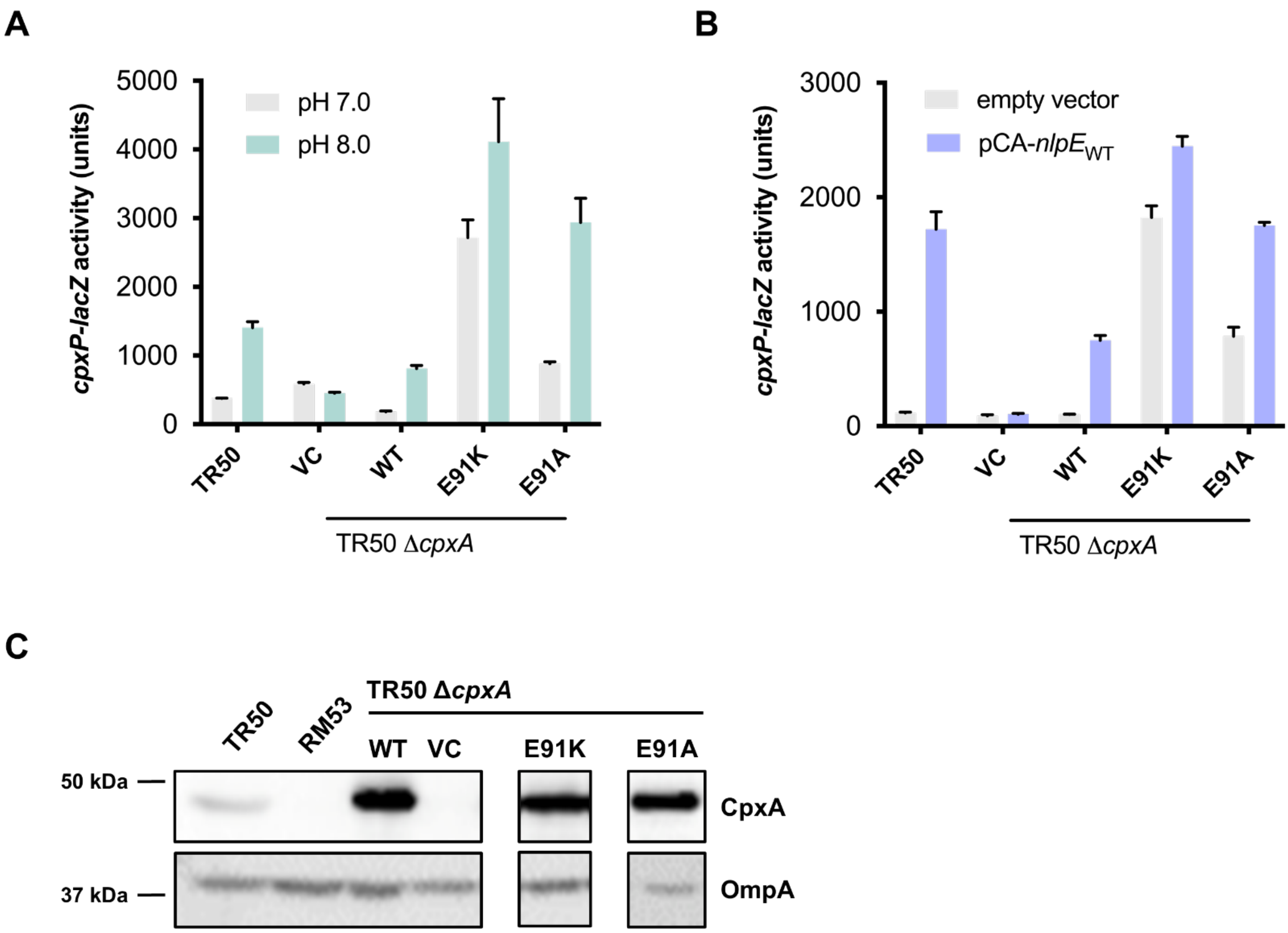

Figure S8. Ability of plasmid-borne CpxA E91K and E91A variants to sense **(A)** alkaline pH and **(B)** NlpE overexpression. **(C)** shows the expression level of D113K compared to WT CpxA by Western blotting. Shown are mean with standard deviation of three replicates from three independent experiments.

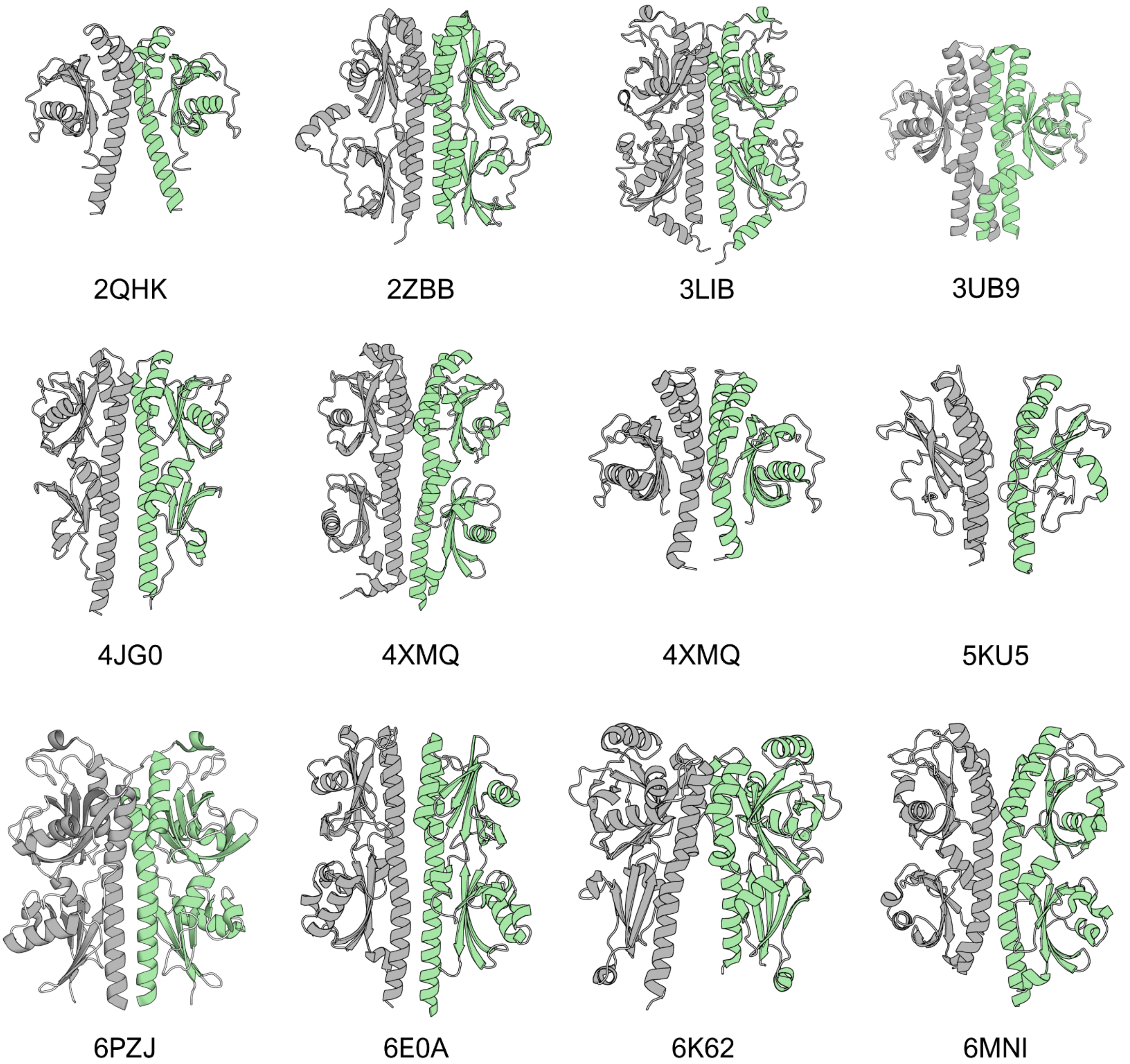

Figure S9. More PAS domain dimer hits of $CpxA_{SD}$. Each monomer is represented as a different colored chain (grey vs green). Protein Database (PDB) codes for each structure are listed below.

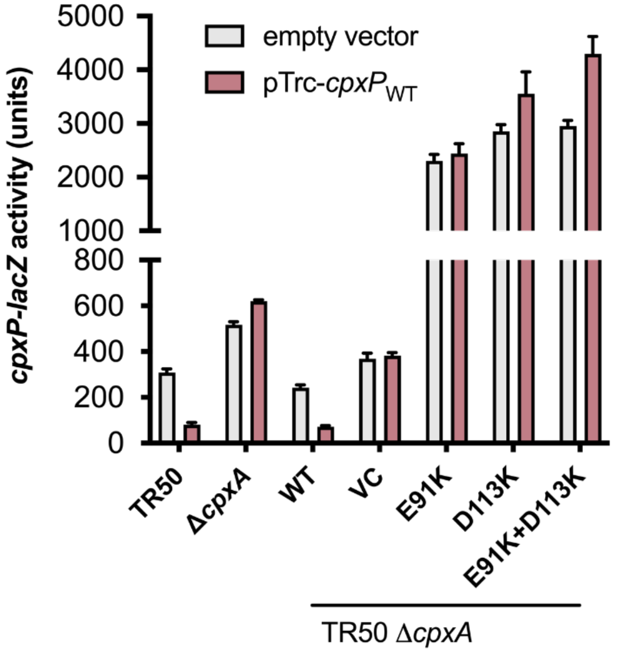

Figure S10. The ability of hyperactivated CpxA variants to sense CpxP overexpression. CpxP was induced from plasmid pTrc-*cpxP* with 0.1 mM IPTG for 2 hours after cells reached mid-log phase. The activity of a *cpxP-lacZ* reporter was used to measure activation of CpxA. Indicated CpxA variants were expressed from plasmid pK184. Shown are mean with standard deviation of three replicates from three independent experiments.

A

	...[EQH]	...[DN]	Other	Total
P...	93	6	56	155
[STDN]...	42	9	21	72
[QE]...	24	4	7	35
Other	45	14	0	59
Total	204	33	84	321

B

	...[EQH]	...[DN]	Other	Total
P...	0	0	0	0
[STDN]...	3	0	63	65
[QE]...	1	0	109	110
Other	16	2	128	146
Total	20	2	299	321

Figure S11. Conservation of N-capping motifs in *cpxA* sequences. **(A)** shows sequence motifs that are present at the N-cap site. **(B)** shows the lack of the presence of N-capping motifs in sequences immediately following the N-cap position in CpxA-SD$_{EC}$ and CpxA-SD$_{Vib}$.

This readme is for analyzing the dali results of CpxA.

Required Files:

Original Files:

parseDali.py (makes the hit dictionary) (https://pastebin.com/wmcR46k5)

makePymolSessions.py (performs search) (https://pastebin.com/UHC0xXzZ)

Internet Scripts:

https://pymolwiki.org/index.php/AngleBetweenHelices

http://pymolwiki.org/index.php/RotationAxis

Installation:

For ease of use all required files should be in the same directory. Files in other directories, such as the pymolwiki scripts, can be imported via the sys.path.insert line in makePymolSessions.py.

Execution:

This script requires pymol to be downloaded. Edit the input lines in the makePymolSessions.py file (searchPDB, readFile, writeDir) to the desired file paths and optionally modify any of the search parameters (a3HelixDistanceCutoff, etc.). Run the makePymolSessions.py file via

pymol -cr makePymolSession.py
